# Profiles of Ecosystemic Resilience and Risk: American Indian Adolescent Substance Use during the First Year of the COVID-19 Crisis

**DOI:** 10.3390/ijerph191811228

**Published:** 2022-09-07

**Authors:** Meghan A. Crabtree, Linda R. Stanley, Randall C. Swaim, Mark A. Prince

**Affiliations:** Tri-Ethnic Center for Prevention Research, Colorado State University, Fort Collins, CO 80523, USA

**Keywords:** COVID-19, resilience, American Indian adolescents, latent profile analysis, substance use

## Abstract

The COVID-19 pandemic has caused an unprecedented disruption to the lives of American Indian (AI) adolescents. While reservation-area AI youth already have a higher risk of substance use (SU) compared to their non-AI peers, COVID-19 stressors likely exacerbated this risk. However, COVID-19-specific and general resilience factors may have buffered against increased SU over the course of the pandemic. Using a person-centered, ecosystemic framework of resilience, we used latent profile analysis to identify ecosystemic resilience profiles indicated by general and COVID-19-specific risk and resilience factors, then examined inter-profile changes in alcohol and cannabis use after the onset of the COVID-19 pandemic from the spring of 2020 to the spring of 2021. The sample was 2218 reservation-area AI adolescents (7–12th grade; schools = 20; *M*_age_ = 15, *SD* = 1.7; 52% female). Four profiles emerged: Average Risk and Resilience, High Resilience, Low Resilience, and High Risk. Adolescents with a High-Risk profile demonstrated increases in alcohol and cannabis use, while High Resilience youth demonstrated decreases. These findings support the hypothesized COVID-19-specific ecosystemic resilience profiles and the application of a person-centered ecosystemic framework to identify which AI adolescents are most likely to experience substance use changes during a life-altering crisis like COVID-19.

## 1. Introduction

The onset of the COVID-19 pandemic was an unprecedented and ongoing traumatic disruption to almost every domain of adolescents’ daily lives [1,2,3]. However, this disruption may have had disproportionate consequences for American Indian (AI) adolescents [4,5], particularly reservation-area youth—a population that already experienced significant adversity prior to the onset of COVID-19 [6]. Despite these adversities, AI reservations, families, and culture provide numerous strengths and resources to AI adolescents that mitigate maladaptive health behaviors and promote resilience. Applying an ecosystemic resilience model within reservation-area AI youth [7,8], this study used a person-centered approach to better understand which AI adolescents experienced changes in their substance use following the onset of COVID-19.

### 1.1. Impact of COVID-19 on American Indian Communities

The term “American Indian” is commonly used in the literature to refer to the indigenous peoples of the contiguous United States. We recognize, with respect, several names/terms refer to the diverse collection of indigenous communities within and outside of the United States. We use American Indian here to allow for comparison to previous research. 

AI communities were severely impacted after the onset of COVID-19 in 2020, with rates of illness, hospitalizations, and death substantially higher than the general population [9]. The APM Research Lab reported that by 3 March 2021, American Indians/Alaskan Natives had the highest mortality rates nationwide, with 1 in 390 deaths, compared to 1 in 665 White Americans [10]. Furthermore, the impact of the pandemic within AI communities reinforced long-standing socioeconomic and health inequities, causing additional hardship to many living on AI reservations [11,12,13]. AIs also experienced disproportionate impacts on their mental health. A 2021 poll by NPR, the Robert Wood Johnson Foundation and the Harvard T.H. Chan School of Public Health found that 74% of AIs and Alaskan Natives (compared to 52% of White Americans) reported that someone in their household was struggling with depression, anxiety, stress, and problems sleeping [14]. 

It is important to emphasize that AI communities have shown powerful resilience in the face of historical injustices, including colonization, displacement, intergenerational transmission of historical trauma, and systemic cultural genocide [15,16]. This communal resilience is illustrated in the swift proactive responses tribal governments took to keep their communities safe. For example, many tribal governments implemented strong viral containment measures that surpassed those of adjacent states and non-tribal communities [17]. Furthermore, community members engaged in collective efforts to reduce transmission; for example, vaccine uptake and acceptance among members of the Navajo Nation was extremely high. By 5 August 2021, daily vaccination rates on the Navajo Nation were at 72,000 per 100,000 of the total population in IHS jurisdictions [18]. Nevertheless, the impact of COVID-19 on AI communities was, and continues to be, profound. There is a significant gap in knowledge among researchers and public health officials, however, regarding the impacts of COVID-19 on reservation-dwelling AI adolescents’ health risk behaviors.

### 1.2. Impact of COVID-19 on AI Adolescents Health Risk Behaviors

The impact of COVID-19 on children and adolescents has been severe [19,20]. The effects of lockdowns, abrupt changes to remote learning, and the resulting social isolation has led to new or intensified mental health problems. In one prospective study conducted by Magson and colleagues, for example, adolescents reported significant increases in depression and anxiety since the onset of COVID-19, with academic challenges and family conflict predicting these increases [21]. However, the same study also found evidence that home quarantining and perceived social connectedness were protective against the psychosocial impacts of COVID-19. 

Although there is limited research addressing the impact of COVID-19 on AI adolescents, a rapid assessment study of urban AI adolescents conducted in early 2020 suggests they were similarly impacted by the life disruptions caused by COVID-19 [22]. This study found heightened levels of anxiety (18%), depression (22%), and traumatic stress (28%), but also relatively low levels of substance use. Additionally, the study found evidence of resilient adaption to the social consequences of COVID-19; many urban AI youth reported low levels of family conflict, high levels of family cohesion, and high levels of participation in traditional practices to cope with stress. 

We are aware of no similar data regarding the impact of COVID-19 on reservation-area AI adolescents’ changes in substance use. However, reservation-area AI youth, on average, demonstrate higher rates of substance use compared to all other ethnic and racial demographics in the United States [23,24]. In a recent epidemiological study, AI 8th graders reported 3.5 times more frequent binge drinking and 5.4 times greater marijuana use in the last 30 days compared to U.S. adolescents [6]. Thus, AI youth experienced significant substance use disparities prior to the onset of COVID-19. However, there is limited research addressing how AI adolescents’ substance use behaviors have changed during the first year of COVID-19, and which AI adolescents were most likely to experience these changes. 

### 1.3. Ecosystemic Resilience Model

An ecosystemic resilience model (ERM) provides the underlying framework for the present study [7,8,25]. Despite their heightened exposure to risk and adverse experiences, many reservation-area AI youth either do not use drugs or alcohol, or they delay initiation until they are older. Indeed, AI communities, families, and culture imbue numerous assets and resources that can reduce or even counteract substance use risk. Ecosystemic theories define resilience as the process by which individuals navigate existing assets and negotiate the resources they need to adapt in response to stressful or traumatic conditions [8,26,27,28]. These theories generate useful models for investigating substance use risk and resilience among AI youth, as they take an ecological and culturally mindful approach to understanding why a large proportion of otherwise “vulnerable” youth do not go on to develop negative outcomes [29]. Another strength of ERMs is their emphasis on the dynamic nature of “resilience”, in that the form resilience takes is expected to differ based on situational and cultural contexts [30]. Finally, rather than characterizing risk and resilience as individual characteristics, an ecosystemic model defines risk and resilience as a complex process that occurs across multiple systems, acknowledging the inherent interplay between individual, interpersonal, and environmental systems in promoting, or inhibiting, adolescents’ resilience [31].

With respect to resilience promoting factors, ERM researchers typically make a distinction between assets (positive internal factors, e.g., a sense of cultural identity) and resources (positive external factors, e.g., having a close-knit family). Likewise, for risk factors that may inhibit resilience [32], ecosystemic models distinguish vulnerabilities (maladaptive internal factors, e.g., high negative affect) from adversity (challenging external factors, e.g., stressful life events). Thus, within an ERM, assets and vulnerabilities represent intrapersonal, individual-level factors, while resources and adversities reflect external, socio-environmental factors, all of which promote or interfere with adolescents’ resilience within certain contexts.

ERMs are particularly applicable to understanding person-level indicators of risk and resilience in the context of the COVID-19 crisis. COVID-19-related stressors, such as psychological distress, health anxiety, and maladaptive coping may increase substance use risk among certain adolescents, either by undermining their access to assets and resources that promote positive adaptation to stressful conditions, or by producing additive effects of vulnerability and adversity that increase adolescents’ substance use risk [33,34]. On the other hand, COVID-19-relevant assets and resources, such as positive coping to deal with COVID-19 stress or having a close-knit family, may buffer or compensate for the negative impacts of the COVID-19 crisis [35,36]. 

Indeed, findings from a survey of Canadian adolescents’ substance use from pre- to post-COVID-19 pandemic highlights the multifaceted impact that the pandemic had on the substance use outcomes of different adolescents. While the percentage of youth who reported using substances dropped after the onset of COVID-19, those adolescents who reported using prior to COVID-19 did so significantly more frequently [34]. These results suggest complex, potentially competing pathways by which the onset of COVID-19 may have reduced substance use rates among some AI adolescents, while worsening the severity of substance use among others.

### 1.4. Present Study 

Using a person-centered approach, the present study applied an ERM to explore the complex impact of COVID-19 on changes in AI adolescents’ substance use after the onset of the pandemic from the spring of 2020 to the spring of 2021. As opposed to a variable-centered approach, which assumes all observations of a phenomenon are drawn from one population, a person-centered approach identifies sub-populations of individuals who share a similar pattern of characteristics [37,38,39]. As person-centered approaches to understanding ecosystemic resilience are rare within the extant research—particularly as applied to health-risk behaviors like substance use—the present study informs ERMs by identifying patterns of internal and external risk and resilience that characterize subsets of AI adolescents who are most likely to engage in substance use behaviors during a wide-spread and ongoing crisis like COVID-19. Latent profile analysis (LPA) is one such person-centered analytical approach intended to recover latent profiles, or unobserved latent mixture components within observable data, defined by continuous observable variables also called indicators [40]. In the present study, LPA was used to identify distinct patterns, or profiles of ecosystemic resilience indicated by general and COVID-19-specific assets, vulnerabilities, resources, and adversities. After identifying these latent ecosystemic profiles of resilience within our sample of AI youth, we examined inter-profile differences in AI youth’s self-reported changes in cannabis and alcohol use over the course of the first year of the COVID-19 pandemic. 

#### Hypotheses

First, based on our application of the ERM, we predicted that distinct latent profiles of risk and resilience would emerge, indicated by higher or lower than average vulnerabilities and adversities, as well as higher or lower than average assets and resources. Specifically, we anticipated the emergence of a high resilience profile characterized by high levels of internal assets and access to external resources, in addition to moderate to low levels of adversity. Additionally, we anticipated the emergence of a high-risk profile characterized by high levels of internal vulnerabilities and experiences of external adversity, with moderate to low levels of assets and resources 

Second, we predicted that AI students with the most likely profile membership indicated by high levels of vulnerabilities or adversities would report increases in substance use, on average, after the beginning of COVID-19. In contrast, students with the most likely profile membership indicated by high levels of assets and resources would report either decreases or no change in their substance use since the beginning of the pandemic. 

## 2. Materials and Methods

### 2.1. School Sampling and Recruitment

Study data were collected from 20 schools (17.1% of schools initially recruited) that participated in the Our Youth Our Future (OYOF) study during the spring 2021 semester. The OYOF study collects nationally representative substance use data for reservation-area middle and high school students on an annual basis. During spring 2021, COVID-19-related psychosocial and behavioral measures were added to the survey to better understand the impact of COVID-19 on reservation-dwelling AI students’ well-being and substance use since the start of COVID-19. Specific identities of tribes and reservations were kept confidential. 

### 2.2. Participants

All students enrolled in each participating school on survey administration day were eligible to participate. Approximately 17.1% of schools that were contacted for recruitment participated in the spring 2021 survey. The response rate at the school-level was below past rates of OYOF school participation. Wide-scale changes to school procedures, settings, and curriculum because of COVID-19 social distancing measures during the spring of 2021 presented several recruitment challenges, especially in those areas most impacted by COVID-19. In total, 3847 students in grades 6–12 were surveyed across the 20 participating schools. Participants represented 60.4% of eligible students in these schools on average, with 70% of schools surveying 60% or more of their eligible students. The present study includes only AI adolescents (*n* = 2218) attending 7–12th grades (*M*_age_ = 15, *SD* = 1.7; 52% female). 

### 2.3. Procedures

All study procedures were approved by the investigating institutional IRB, as well as participating school and tribal boards where appropriate. Approximately 3 weeks prior to the scheduled survey, letters were sent to parents of enrolled middle and high school students with a survey description and instructions for opting their child out of the survey (<1% of participants were opted out). This information was also posted on local media sites where parents were likely to see it. Students completed the survey via Qualtrics during school hours. Student who did not participate in the survey were typically absent on the day of survey administration. On average, students spent approximately 35 min completing the survey.

Due to varying COVID-19 operating procedures, eleven schools were operating concurrently with some students remote and some in-person; six schools used a hybrid approach where students were part-time remote and part-time in-person; and three schools were in-person only. Schools could survey remote students if a faculty member was virtually present during survey administration. Approximately 69.5% of the total OYOF sample and 60.2% of the study sample completed the survey on campus. 

### 2.4. Measures 

#### 2.4.1. Indicators of Latent Profiles 

Indicators are categorized below according to the risk and resilience factors of the ERM described in the introduction; that is, internal vulnerabilities and assets, and external adversities and resources. COVID-19-specific psychosocial measures in the OYOF survey came from multiple sources, including the 2020 Monitoring the Future survey, the CASPE Adolescent Self-Report Survey [41], and the Environmental Influences on Child Health Outcomes COVID-19 Questionnaire-Child Self-Report [42]. Some items were modified to reduce cognitive load and accommodate students with lower reading levels in consultation with a literacy expert. Except where otherwise specified, composite variables were created for each latent profile indicator below by averaging across items. Profile indicators were standardized prior to inclusion in each LPA. Items comprising all indicators are listed in Table A1 in Appendix A.

##### Internal Vulnerability

Three measures of COVID-19-specific internal vulnerabilities were included in the study. Six items assessed the extent to which students were anxious about COVID-19-related health outcomes for themselves and others (COVID-19 health anxiety Cronbach’s α (hereafter α) = 0.91); ten items assessed changes in negative affect since the onset of the pandemic in 2020 (COVID-19-specific change in negative affect; α = 0.92). Four items measured frequency of students’ maladaptive coping behaviors in response to COVID-19 stress, focusing on drug use (COVID-19-specific maladaptive coping; α = 0.81). 

##### Internal Assets

Seven measures of internal assets were included, with three being COVID-19-related and three being non-COVID-19-related. Two measures assessed adaptive coping mechanisms. COVID-19-specific prosocial coping was measured with five items (α = 0.62) while COVID-19-specific distracted coping was measured with three items (α = 0.62). Three items measured school engagement during the pandemic (α = 0.76). Trait resilience was measured using the ten-item Connor–Davidson Resilience Scale [43] (CD-RISC; Campbell-Sills and Stein, 2007), validated in both minority adolescent and AI populations [44,45] (α = 0.89). Racial–ethnic identity was measured using the Racial–Ethnic Identity Scale [46], which consists of three dimensions: embedded achievement (“If I am successful, it will help the American Indian community”), connectedness (“I feel that I am part of the American Indian community”), and awareness of racism (“Some people will treat me differently because I am American Indian”). Cronbach’s α’s were 0.78, 0.80, and 0.71, respectively. 

##### External Adversity 

Stressful life events were measured using a modified version of the Schedule of Recent Experiences [47], which asks students to indicate whether they experienced one or more of seven specific stressful life events over the prior 12 months. A composite variable was created by summing across items. Three items measured school challenges during the COVID-19 pandemic (COVID-19-specific school challenges; α = 0.83).

##### External Resources

Three items measured family closeness during the COVID-19 pandemic (Cronbach’s α = 0.71), while two items measured peer closeness during the COVID-19 pandemic (*r* = 0.57). 

#### 2.4.2. Distal Outcomes

Distal outcomes of latent profile assignment included self-reported changes in cannabis smoking and cannabis edible use, as well as alcohol use “after COVID-19 began” in 2020. The four change items were measured on a 5-point scale: “Decreased a lot” (−2), “Decreased a little” (−1), “No change” (0), “Increased a little” (1), and “Increased a lot” (2). 

### 2.5. Analytical Procedures 

To reduce survey fatigue, a planned missingness design was employed for approximately one-third of survey items, including most of the profile indicator items reported above. However, all participants completed demographics, COVID-19-specific substance use measures, and the coping and stressful life events scales. The remaining items were split into three forms. Participants were randomly assigned to complete two of the three forms, such that two-thirds of participants were able to respond to any given planned missingness item. Because respondents were randomly assigned to each form, planned non-response data is considered missing completely at random [48]. Additionally, the order of items was randomized to reduce systematic missingness [49]. By default, Mplus excluded cases with variables missing on all variables or all distal outcomes (*n* = 216), bringing the final analytical sample to 2002. 

OYOF data used in the present study were analyzed using Mplus version 8.6 [50]. A series of LPAs were conducted using the maximum likelihood estimator with robust standard errors (MLR), which uses full information maximum likelihood to account for data missing at random (MAR) within the profile indicators [51]. We employed a sandwich estimator within Mplus to adjust standard errors to account for non-independence of students clustered within schools [52]. 

One to five plausible solutions were specified using an iterative modeling process at each stage in our analytical procedures to determine the optimum number of profiles for any given model. The optimal number of profiles was determined using a combination of statistical fit criteria and classification quality indices, specifically the Sample-size Adjusted Bayesian Information Criteria (SABIC; lower values representing better fit), entropy (≥0.80 is ideal), successful loglikelihood replication, and smallest class size (>5% of cases for replication purposes), average latent class probabilities, and substantive interpretability of the profiles based on ecosystemic theories of resilience. We note that a simulation study has shown SABIC to be similarly reliable and robust as the bootstrap likelihood ratio test and the Lo–Mendel–Ruben in identifying the best-fitting model within nested samples [53]. 

Students were collapsed into two grade groups: 7–9th grade and 10–12th grade. Analytical procedures were conducted in three stages. First, one to five plausible solutions were tested incrementally for each grade group (7–9th vs. 10–12th) and sex (male vs. female), to obtain four potentially optimal solutions for each group. Second, once a plausible solution was determined for each group, we performed two multigroup LPAs, constraining class-specific item response probabilities to be equal between male and female adolescents, and 7–9th graders and 10–12th graders, to test for measurement equivalence between the latent profile solutions [54]. Third, after determining measurement equivalence, we used two 3-step approaches specified within Mplus [55] to examine (1) sex as a covariate of latent profile membership (R3STEP), and (2) retrospectively reported changes in alcohol use, cannabis smoking, and cannabis edible use after the start of the COVID-19 pandemic (BCH). 

## 3. Results

Table 1 provides correlations between the latent profile indicators and distal outcomes. Intercorrelations were mostly small across ecosystemic indicators, and small–moderate within ESR framework categories.

### 3.1. Latent Profiles of Ecosystemic Resilience

After taking into consideration all combinations of classification indicators, a four-model solution was suggestive of the optimal solution for each sex and grade group. Next, we conducted two multigroup invariance tests of measurement equivalence for sex and grade-group separately to ascertain whether class-specific conditional probabilities between each group were equivalent (i.e., measurement equivalence). Based on the sample size adjusted BIC and negative loglikelihood test difference test (*-LLΔ*) with scaling correction for robust maximum likelihood (*cf*) [56], the model constraining the class-specific item response probabilities to be equal between male and female adolescents [*-LL* (130) = 34,748.975; *cf* = 1.9789; *SABIC* = 70,073.050] did not demonstrate a worse fit compared to the unconstrained model [*-LL* (133) = 34,744.271; *cf* = 2.0249; *SABIC* = 70,076.913], with *-LLΔ* (3) = 2.3414, *p* = 0.50. However, based on these same criteria, measurement equivalence was not obtained across grade-groups, indicating that class-specific conditional response probabilities were not equivalent between 7–9th graders and 10–12th graders. Thus, a separate LPA was conducted for each grade group at the third stage of our analysis.

As mentioned above, the four-profile solution was the ideal solution for each grade group collapsing across sex, indicated by a combination of SABIC, entropy, profile sample-sizes, probability classification, and substantive interpretation (see Table 2). Note that entropy was slightly lower than the general rule-of-thumb benchmark for the four-profile solution among 7–9th graders (0.796), whereas a three-profile solution was slightly higher than the benchmark (0.801). However, taking into consideration all statistical and theoretical criteria, particularly the similarity of fit and classification between a three vs. four-profile solution, we felt that the four-profile solution yielded a more theoretically consistent and distinct pattern of ecosystemic resilience profiles based on the standardized indicator means. 

**Figure 1:**
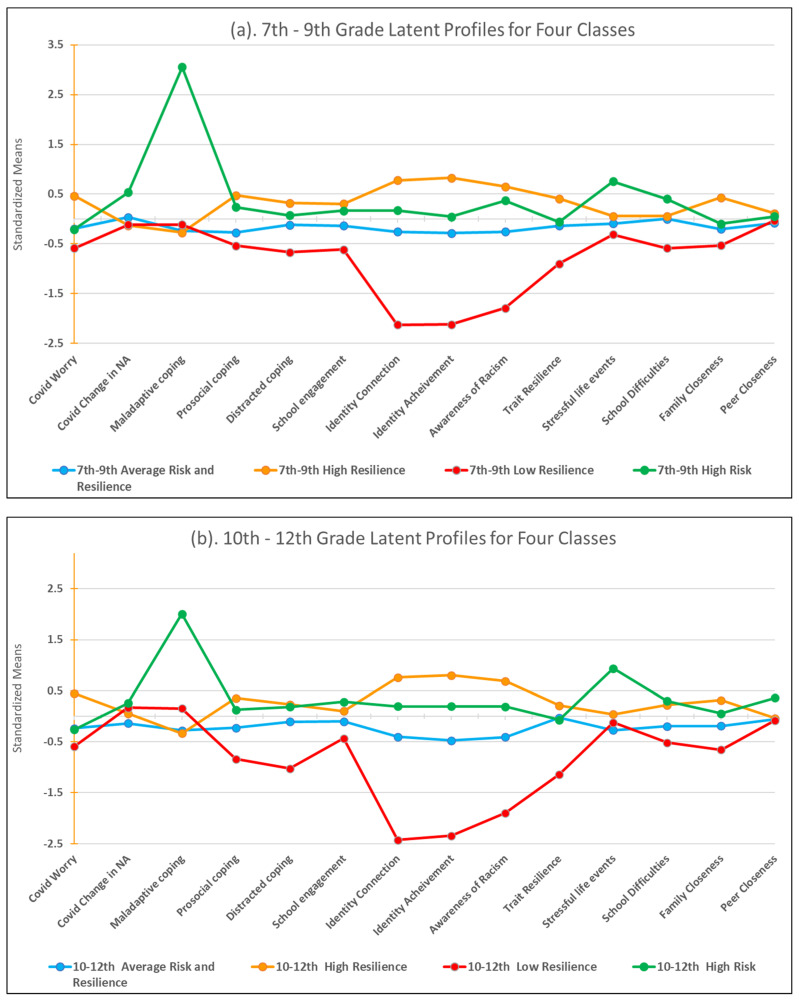
(**a**) 7–9th Grade Latent Profiles for Four Classes; (**b**) 10–12th Grade Latent Profiles for Four Classes.

Figure 1a,b provides the pattern of standardized mean scores for each of the four profiles across indicators for 7–9th graders and 10–12th graders, respectively. To illustrate the pattern of profile differences between ecosystemic indicators, significant inter-profile differences in the means of each indicator are provided in Table 3. Indicator means were compared within Mplus using the model constraint command to test the significance of the mean difference of each indicator compared between each of the four profiles. 

#### Latent Profiles

Total and profile-specific sample sizes, descriptives, indicator means, and distal means are provided in Table 3 for each grade group. Theoretical interpretation of the four profile patterns was grounded within the ERM framework. The largest profile, describing approximately 50% of the 7–9th grade sample and 43% of the 10–12th grade sample, was labeled Average Risk and Resilience. This profile characterized AI adolescents with average levels of vulnerabilities, assets, adversities, and resources. 

The next largest profile, representing approximately 35% of the 7–9th sample and 38% of the 10–12th grade sample was labeled High Resilience. This profile characterized adolescents who reported higher levels of assets and resources relative to the other profiles. The third profile, labeled Low Resilience, represented approximately 8% of the 7–9th sample and 6% of the 10–12th grade sample. This profile characterized adolescents who reported significantly lower levels of all psychological and behavioral assets compared to the other profiles. Finally, the fourth profile, labeled High Risk, described approximately 7% of the 7–9th grade sample and 12% of the 10–12th grade sample. Adolescents with this profile were most notably characterized by high psychological and behavioral vulnerabilities, as well as external adversities.

### 3.2. Auxiliary Analyses

#### 3.2.1. Sex 

The multinomial logistic coefficients (log-odds), *p*-values, odds ratios, and 95% confidence intervals for the odds of membership in each profile for male vs. female adolescents are provided below, which were derived from the R3STEP auxiliary three-step approach available within Mplus [55]. The odds ratios represent the strength of the relationship between the auxiliary covariate (i.e., being female) and membership in a designated profile compared to a referent profile. The proportion of male and female adolescents within each latent profile are provided in Table 3. 

##### 7–9th Grade Profiles 

Significant differences in the odds of profile membership were found for the Low Resilience profile relative to the other three profiles. Female adolescents in the 7–9th grade were approximately 3–5 times more likely than male adolescents to have High Resilience (*b* = 1.13, *p* = 0.001, OR = 3.1, 95% CI [1.6, 5.9]), Average Risk and Resilience (*b* = 1.17, *p* = 0.001, OR = 3.2, [1.6, 6.5]), or High Risk (*b* = 1.57, *p* < 0.001, OR = 4.8, [2.9, 8.1]) profiles, compared to the Low Resilience profile. 

By contrast, adolescents’ sex did not discriminate their odds of membership in the High Risk profile compared to either the Average Risk and Resilience (*b* = –0.40, *p* = 0.189, OR = 0.67, 95% CI [0.37, 1.22]), or High Resilience profiles (*b* = –0.44, *p* = 0.183, OR = 0.64, [0.34, 1.23]), nor for the Average Risk and Resilience profile compared to the High Resilience profile (*b* = −0.041, *p* = 0.827, OR = 0.96, [0.67, 1.38]). Thus, for 7th and 9th graders, AI adolescents’ sex only discriminated their odds of membership in the Low Resilience profile, with female adolescents least likely, and male adolescents most likely to be characterized by Low Resilience. 

##### 10–12th Grade Profiles

Adolescents’ sex discriminated their odds of membership in both the Low Resilience profile and Average Risk and Resilience profiles. Similar to 7–9th grade female adolescents, those in 10th–12th grade were approximately 2–5 times more likely than male adolescents to be characterized by the Average Risk and Resilience (*b* = 0.86, *p* < 0.001, OR = 2.4, 95% CI [1.6, 3.5]), High Risk (*b* = 1.24, *p* < 0.001, OR = 3.5 [2.0, 5.8]), or High Resilience (*b* = 1.59, *p* < 0.001, OR = 4.9, [3.1, 7.8]) profiles compared to the Low Resilience profile. Thus, once again, female adolescents were the least likely, and male adolescents were the most likely, to be characterized by a Low Resilience profile.

Furthermore, female adolescents were twice as likely to have a High Resilience profile compared to the Average Risk and Resilience profile (*b* = 0.72, *p* < 0.001, OR = 2.1[1.4, 3.0]). However, adolescents’ sex did not discriminate their odds of profile membership in the High Risk profile compared to High Resilience (*b* = 0.35, *p* = 0.295, OR = 1.41, [0.74, 2.71], or Average Risk and Resilience (*b* = −0.38, *p* = 0.061, OR = 0.69, [0.46, 1.02] profiles. 

#### 3.2.2. Distal Outcomes: Mean Comparisons of Substance Use Changes between Profiles 

Equality of mean changes in alcohol use, marijuana smoking, and marijuana edible use across ecosystemic profiles was tested using the 3-step BCH auxiliary procedure available within Mplus. Overall, the absolute magnitude of change in AI adolescents’ alcohol use, cannabis smoking, and cannabis edible use was small, though there were some inter-profile differences. Results are illustrated in Table 4, and significant differences across profiles are discussed below. 

##### Change in Alcohol Use

1. 7–9th Grades

The omnibus test was not significant, with χ^2^(3) = 4.34, *p* = 0.228, indicating no profile differences in alcohol use change among 7th–9th graders.

2. 10–12th Grades

The omnibus test was significant, with χ^2^(3) = 34.45, *p* < 0.001. Adolescents with a High Risk profile reported the greatest increases in alcohol use (*M* = 0.374), relative to those with the other three profiles: Low Resilience (*M* = −0.08, *p* = 0.002), Average Risk and Resilience (*M* = −0.11, *p* < 0.001), and High Resilience (*M* = −0.08, *p* < 0.001). Interestingly, adolescents with a High Resilience, Low Resilience, or Average Risk and Resilience profile reported decreases in alcohol use, with no significant differences in change between profiles (High Resilience vs. Low Resilience, *p* = 0.981; High Resilience vs. Average Risk and Resilience, *p* = 0.074; Average Risk and Resilience vs. Low Resilience, *p* = 0.651). 

##### Change in Cannabis Use (Smoking)

1. 7–9th Grade

The omnibus test was significant, with χ^2^(3) = 20.21, *p* < 0.001. Again, adolescents with the High Risk profile reported significantly greater increases in cannabis smoking (*M* = 0.41) relative to adolescents with any other profile: Low Resilience (*M* = 0.07, *p* = 0.031); High Resilience (*M* = −0.12, *p* < 0.001); and Average Risk and Resilience (*M* = −0.03, *p* = 0.006). Additionally, adolescents with the Low Resilience profile reported significantly greater increases in cannabis smoking relative to those with a High Resilience profile (*p* = 0.003), but not those with the Average Risk and Resilience profile (*p* =0.054). Although adolescents with the High Resilience profile reported larger average decreases in cannabis smoking than those with the Average Risk and Resilience profile, the difference was not statistically significant (*p* = 0.074).

2. 10–12th Grades

The omnibus test was significant, with χ^2^(3) = 125.33, *p* < 0.001. Again, adolescents with the High Risk profile reported the greatest increases in cannabis smoking (*M* = 0.53) compared to all other profiles: Low Resilience (*M* = −0.06, *p* = 0.004), Average Risk and Resilience (*M* = −0.05, *p* < 0.001), and High Resilience (*M* = −0.05, *p* < 0.001). Similar to their changes in alcohol use, adolescents with High Resilience, Low Resilience, and Average Risk and Resilience profiles all reported similar decreases over the first year of the COVID-19 pandemic (High Resilience vs. Low Resilience, *p* = 0.956; High Resilience vs. Average Risk and Resilience, *p* = 0.992; Average Risk and Resilience vs. Low Resilience, *p* = 0.945). 

##### Change in Cannabis Use (Edibles)

1. 7–9th Grades

The omnibus test was significant, with χ^2^(3) = 16.84, *p* = 0.001. On average, adolescents with the High Risk profile reported significantly greater increases in edible use (*M* = 0.22) compared to those with the Average Risk and Resilience (*M* = −0.07, *p* = 0.006) and the High Resilience profiles (*M* = −0.10, *p* < 0.001), but not the Low Resilience profile (*M* = 0.01, *p* = 0.065). Adolescents with High Resilience, Low Resilience, or Average Risk and Resilience profiles demonstrated no significant differences in their magnitude of change over the first year of the pandemic (High Resilience vs. Low Resilience, *p* = 0.234; High Resilience vs. Average Risk and Resilience, *p* = 0.508; Average Risk and Resilience vs. Low Resilience, *p* = 0.326).

2. 10–12th Grades

The omnibus test was not significant, with χ^2^(3) = 3.63, *p* = 0.304, indicating no profile differences in the magnitude of change in use of edible cannabis use among 10th–12th graders.

## 4. Discussion

The study findings support the hypothesized COVID-19-specific ecosystemic resilience profiles. Likewise, the findings support the application of a person-centered ecosystemic framework to identify which AI adolescents are most likely to experience substance use changes during a life-altering crisis like COVID-19, based on ecosystemic factors of risk and resilience.Four profiles emerged for both grade groups in the present sample of AI youth: (1) an Average Risk and Resilience profile characterized by moderate or mixed levels of all ecosystemic indicators (43–50%); (2) a High Resilience profile characterized by relatively high internal assets and external resources (35–38%); (3) a Low Resilience profile characterized by relatively low internal assets and external resources (6–8%); and (4) a High Risk profile characterized by high internal vulnerabilities and external adversities (7–12%). AI male adolescents in both grade groups were most likely to be characterized by Low Resilience relative to any other profile. Finally, inter-profile differences in alcohol and cannabis use changes were limited. Adolescents with a High Risk profile reported the largest changes (increases) in alcohol and cannabis use during COVID-19—depending on the grade group. However, there were no consistent inter-profile differences in substance use changes between AI adolescents most likely to be characterized by the other three profiles. Our findings support a person-centered approach to understanding ecosystemic resilience and underline how relatively complex patterns of socio-environmental resources and adversity, in conjunction with psychological assets and vulnerabilities, may predict increased or decreased substance use among reservation-area AI youth exposed to an unprecedented and large-scale crisis like COVID-19. 

### 4.1. Ecosystemic Resilience Profiles

The emergence of High Resilience, Low Resilience, and High Risk profiles supports our first hypothesis that a profile characterized by high assets and resources with moderate adversity and vulnerability, and a profile characterized by high adversity and vulnerability with moderate to low assets and resources, would derive from the indicators based on the ERM; however, the pattern of indicators within each profile was complex. For example, AI youth with a High Resilience profile did not necessarily report lower levels of vulnerability or adversity, nor did youth with a Low Resilience profile report higher levels of vulnerability and adversity. Furthermore, youth characterized by the High Risk profile reported moderately high levels of many assets and resources. These findings are consistent with how the extant research conceptualizes resilience, that is, as positive adaption in response to adversity or vulnerability, not the complete absence of these risk factors [57]. Furthermore, ecosystemic perspectives of resilience have emphasized that risk and resilience are not mutually exclusive; rather, they reflect dynamic and context-specific processes contributing to adolescents’ positive or negative behavioral outcomes [8]. Indeed, multisystem frameworks of resilience argue that moderate levels of adversity induce stress inoculation, which promote adaptive functioning in the face of future adversity. On the other hand, heightened levels of adversity and vulnerability can overwhelm existing stress-responsive systems within individuals, leading to a struggle to cope despite pre-existing resilience promoting assets and resources [58]. 

A large proportion of AI adolescents were most likely to be characterized by a High Resilience profile. Thus, a resilient response to the stress of COVID-19 was normative among AI youth, which is broadly consistent with the extant resilience research [59,60,61,62]. Among the assets and resources measured across both grade groups, high levels of family closeness, AI identity connectedness, and AI identity achievement best distinguished the High Resilience profile. Unexpectedly, high levels of COVID-19 health anxiety were also a distinguishing indicator of High Resilience.

Family closeness is a critical resource for promoting resilience among AI youth [30,63], as AI families are the mechanism by which adolescents learn about their cultural beliefs, values, and traditions. From an indigenous perspective, family closeness is an important feature of a resilient family [64,65]. Although large sample-size empirical works addressing COVID-19’s effects on reservation-dwelling AI families are not available, findings from a recent mixed-method rapid-assessment study of risk and resilience among urban AI and Alaska Native youth reported increases in family cohesion and relatively low levels of family conflict during the extended stay-at-home mandates in place after the COVID-19 onset [22]. The present study supports and extends this work by demonstrating that greater levels of family closeness among reservation-dwelling AI youth was a strong indicator of resilience during COVID-19. 

High Resilience youth also reported the strongest feelings of connectedness, embedded achievement, and awareness of racism in relation to their AI identity. Based on theories of social identity [66,67,68], Oyserman and colleagues developed the Tripartite Model of racial–ethnic identity [46], which posits that these three dimensions interact to promote prosocial behaviors and positive self-regard—all strongly linked to resilience across multiple populations [28]. According to the Tripartite model, connectedness, or a positive sense of in-group belonging and racial–ethnic group pride, reinforces the salience of adolescents’ racial–ethnic identity within their self-concepts and strengthens their motivation to act in accordance with their groups’ norms and values—particularly in the face of discrimination. Embedded achievement, or the belief that attainment is a quality of individuals in adolescents’ racial–ethnic group, likewise buffers against the experience of negative stereotyping and acts as a behavioral guide for adolescents’ prosocial enactment of their racial–ethnic identity. Awareness of racism, or an adolescents’ knowledge that others may underestimate their capabilities and undervalue their achievements because of their racial–ethnic identity, has been shown to buffer against the impact of negative expectations and promote persistence in achieving positive outcomes [69]. In particular, awareness of racism (as it is defined within the Tripartite model) enables adolescents to make attributions of negative feedback to discrimination against their racial–ethnic group, rather than to their own perceived capabilities [70]. 

Several studies have demonstrated that a strong racial–ethnic identity, and similar constructs like ethnic pride and cultural identity, are protective against behavioral risk for AI youth [71,72,73]. For example, Kulis and colleagues found that ethnic pride promoted anti-drug norms among AI youth [74], while Baldwin and colleagues found that cultural identification buffered the effect of stressful life events and protected against engagement in risky behaviors via AI adolescents’ relationships with their family and prosocial peers [47]. 

Surprisingly, High Resilience youth also reported the highest levels of anxiety over the impact of COVID-19 on themselves and their loved ones. The fact that High Resilience AI youth also reported high internal vulnerabilities does not contradict prevailing theories of resilience. However, it is possible that, at the person-level, anxiety over COVID-19’s impact on loved ones operated as an internal asset among High Resilience youth. For example, COVID-19-related concern for others has been linked to a sense of altruism and empathy [75], which is positively related to psychological well-being among adolescents [76]. Likewise, given their strong racial–ethnic connectedness, high resilience AI youth may have been more concerned over the health and well-being of those within their respective reservation communities, particularly in light of the severe impacts of COVID-19. 

Supporting this inference, Whitesell and colleagues found a positive relationship between AI adolescents’ community-mindedness and the strength of their AI identities, along with evidence that cultural identity is key to the development of community-mindedness [77]. As such, heightened COVID-19 anxiety may be indicative of greater community-mindedness among AI youth with stronger racial–ethnic identities. Re-categorizing COVID-19-specific health anxiety as an internal asset rather than a vulnerability would provide a more consistent and interpretable pattern of indicators for both the High Resilience and the Low Resilience Profiles, as youth characterized by the latter reported the lowest levels of COVID-19 anxiety and racial–ethnic identity in both grade groups.

Youth in both grade groups most likely to be characterized by a Low Resilience profile reported the lowest levels of all internal assets, particularly school engagement, family closeness, and all three dimensions of racial–ethnic identity. They also reported moderately few stressful life events, maladaptive coping, and fewer school challenges than AI youth most likely to be characterized by the other three profiles. This finding is unsurprising, as resilience, by definition, cannot manifest in the relative absence of vulnerabilities or adverse conditions. Nevertheless, the magnitude of difference in levels of racial–ethnic identity between adolescents with a Low Resilience profile vs. the other profiles is prominent, and suggests Low Resilience youth feel disengaged from their AI culture and disconnected from other members of their AI communities. This disconnection is also illustrated in their low levels of family closeness [65]. 

Unfortunately, it is not possible to determine whether Low Resilience AI youth experienced a reduced sense of racial–ethnic identity prior to COVID-19. However, the onset of COVID-19 held significant social and cultural consequences for AI communities, particularly as shelter-in-place mandates were enacted by tribal governments to mitigate the extremely high rates of COVID-19-related deaths. Although these proactive mandates were necessary for the survival of vulnerable AI communities, prolonged isolation from friends, extended family members, and elders, as well as the inability to engage in cultural traditions and practices outside of their homes, may have negatively impacted the cultural connectedness of reservation-dwelling AI youth—particularly those who were already vulnerable to cultural loss or disconnection [78]. 

Indigenous peoples, including AI adolescents, continue to be subjected to substantial intergenerational trauma caused by colonial policies that disconnect indigenous youth from their families, cultural traditions, and ways of life [79]. It is important to recognize that cultural disconnection is a result of historical injustices that continue to impair many AI adolescents’ access to and engagement with cultural resources that promote resilience [80]. It is inappropriate to conclude that AI youth most likely to be characterized by a Low Resilience profile have an innate character flaw or personal deficiency that is impeding their resilience. Instead, the emergence of a Low Resilience profile in this study underscores a critical need for more research addressing the complex dynamics involved in the relationships between racial–ethnic identity, historical loss, and AI adolescents’ manifested resilience.

AI youth most likely to be characterized by a Low Resilience profile were significantly more likely to be male relative to any other profile across both grade groups. These results may be consistent with prior studies indicating that AI girls experience a greater sense of, or connection to, their AI identities [81,82,83]. For example, using a sample of AI youth attending a Midwest tribal charter school, Graham found evidence indicating that AI boys had lower enculturation scores than AI girls [83]. Whitesell and colleagues found similar results; AI girls within their study reported a stronger sense of AI identity than the AI boys [81]. 

Although colonization disrupted the traditional gender roles of indigenous men and women within their respective cultures, many North American tribes were historically matriarchal, particularly regarding the responsibility of teaching cultural values and traditions to the next generation [84]. Thus, the persistence of these traditions from generation to generation may empower AI girls and adolescents to engage more with their indigenous cultural traditions and values. Furthermore, there is some indication that the cultural losses wrought by European colonization, the consequences of which continue to perpetuate through intergenerational transmission of trauma, have had a distinctly negative impact on cultural engagement among male AI adolescents [82,85,86]. 

Across both grade groups, AI youth most likely to be characterized by a High Risk profile reported significantly more stressful life events in the 12 months prior to taking the survey, and marked levels of maladaptive coping with COVID-19 stress. Moreover, High Risk 7–9th graders reported increases in negative affect and greater school challenges over the course of the COVID-19 pandemic compared to other AI adolescents. Interestingly, high risk AI youth in both grade groups also reported average to moderately high levels of certain assets and resources, such as racial–ethnic identity, prosocial coping, and family closeness. 

High Risk AI adolescents reported a large accumulation of stressful events just before or during the COVID-19 crisis in 2020—an average of 3 out of 7 stressful events, compared to an average of 1–2 among AI youth most likely to be characterized by other profiles. Additionally, the stressful events most commonly reported by High Risk youth may have been qualitatively different than those of their peers. For example, 40% of High Risk AI youth reported having a friend attempt suicide compared to 11–18% of other youth, and 11% reported being in a serious car accident, compared to 3–5% of other youth. For these High Risk adolescents, experiencing an accumulation of stressful life events during an unprecedented, long-term, and life changing crisis such as the COVID-19 pandemic may have overwhelmed the compensatory effects of their existing assets and resources. Prior works also corroborate this potential phenomenon; in their study of the cumulative effects of risk and promotive factors characterizing adolescent resilience, Ostaszewski and Zimmerman found that the effects of cumulative promotive factors on adolescents’ behavioral risk was substantially smaller in magnitude than the effects of cumulative risk factors [87].

A High Risk profile in the context of COVID-19 is not only indicated by accumulated stressful life events, but also by strikingly high levels of substance use to cope with COVID-19 stress (i.e., maladaptive coping behaviors). Notably, High Risk youth and High Resilience youth across both grade groups reported nearly equivalent levels of prosocial and distracted behaviors to cope with stress. In fact, the High Risk and High Resilience profile are illustrated by a similar pattern of indicators, apart from their stressful event accumulation and engagement in maladaptive coping. It appears that, despite engaging in the same prosocial behaviors to cope with stress; the accumulation of stressful life events in addition to engagement in maladaptive coping behaviors undermined High Risk AI adolescents’ utilization of their assets and resources that would support a resilient response to COVID-19 stress [29]. 

### 4.2. Inter-Profile Changes in Cannabis and Alcohol Use following the Onset of COVID-19

The magnitudes of change in alcohol and cannabis use (either decreases or increases) were relatively small across profiles. However, significant inter-profile differences did emerge in partial support of our second hypothesis. Relative to the changes observed among adolescents most likely to be characterized by the other three profiles, High Risk youth reported the largest increases in cannabis smoking during the COVID-19 pandemic across both grade groups. Interestingly, however, there were no significant inter-profile differences in the absolute values of change between the Average Risk and Resilience, High Resilience, and Low Resilience youth in either grade group, except for cannabis smoking among 7–9th graders. High Resilience 7–9th graders reported significant decreases in cannabis smoking relative to Low Resilience youth, who reported small average increases. 

That High Risk youth reported the largest increases in substance use across grade groups over the course of the COVID-19 pandemic is consistent with present study expectations, as is that High Resilience youth reported overall decreases in alcohol and cannabis youth. However, the relative lack of inter-profile substance use differences between High Resilience and Low Resilience profiles was unexpected. 

Notably, High Resilience and Low Resilience AI youth share similarly low levels of maladaptive coping and stressful life events, high levels of which strongly indicate the probability of having a High Risk profile. Thus, levels of maladaptive coping and number of stressful life events may be the primary factors driving the pattern of inter-profile differences in substance use changes observed within the present study. While engagement in prosocial behaviors may enable most youth to maintain resilience in the face of moderate stress, for the High Risk youth in this study, the prosocial coping strategies they employ do not appear to mitigate the impact of their cumulative stressful experiences. From an ecosystemic perspective, resilience is only experienced when the strategy an individual employs to cope with adversity successfully mitigates the impact of cumulative risk [88]. These findings emphasize the importance of understanding why, and under what conditions, adolescents choose to use substances to cope with their stress. The intensity of stress appears to be an important factor in the present study; however, other unmeasured external or internal factors may have also contributed to these person-level differences—such as having easier access to alcohol or cannabis during the COVID-19 pandemic, or greater persistence in efforts to obtain drugs despite decreases in availability [89].

Although the present study fills an important gap in the literature regarding AI resilience and substance use during the COVID-19 pandemic, the findings should be considered in light of their limitations. First, this study relies on a cross-sectional sample of AI youth living on or near reservations; thus, adolescents’ self-reported experiences during the pandemic, as well as changes in substance use behaviors, may be subject to recall error. Second, the three-step approach used to evaluate inter-profile differences in substance use changes as a distal outcome cannot accommodate control variables. Thus, this study does not control for whether adolescents reported using alcohol or cannabis prior to the onset of COVID-19. However, the three-step approach for testing auxiliary covariates and distal outcomes of profile membership overcomes several disadvantages of other approaches by accounting for the potential misclassification that occurs during estimation of the probability of each participant’s membership in each profile [55]. 

Third, though the broader study’s sampling methodology targeted a nationally representative-sample of AI youth attending reservation-serving schools, the impacts of COVID-19 on school operating conditions during the spring of 2021 introduced recruitment and participation challenges, and school participation rates were below past rates of OYOF school participation. Thus, generalizability may be impacted, as participating schools were likely to be better-resourced than non-participating schools. Similarly, attendance rates were low for all surveyed schools; as such, students with regular school attendance were more likely to be surveyed than students without regular school attendance Thus, the impact of COVID-19 on reservation-area AI students’ risk and resilience is likely underestimated. 

## 5. Conclusions

Using an ecosystemic resilience model as the theoretical framework for this study, the primary objective was to identify ecosystemic profiles of resilience indicated by both COVID-19-specific and non-COVID-19-specific risk and protective factors among reservation-dwelling AI youth. With these emergent profiles, we examined inter-profile differences in the extent to which AI adolescents’ alcohol use, cannabis smoking, and cannabis edible use changed during the first year of the COVID-19 pandemic. This study presents two major findings. First, four distinct ecosystemic profiles emerged among 7–9th graders, and 10–12th graders, respectively: an Average Risk and Resilience profile, a High Resilience profile, a Low Resilience profile, and a High Risk profile. While each profile was characterized by distinct levels of ecosystemic internal vulnerabilities and assets, as well as external resources and adversities, only AI youth most likely to be characterized by a High Risk profile reported changes (increases) in alcohol use, cannabis smoking, and cannabis edibles after the onset of COVID-19 that were significantly different from those of the other three profiles. Based on these findings, future works, including strength-based or resilience focused prevention research, should take into consideration the heterogeneity of risk and resilience within the reservation-dwelling AI adolescent population. This study also indicates that implementation of substance use prevention interventions should accommodate the needs of diverse ecosystemic profiles of risk and resilience that emerge within adolescent populations.

## Figures and Tables

**Table 1:** Correlations between Latent Profile Indicators and Distal Outcomes of Substance Use.

Variables	1	2	3	4	5	6	7	8	9	10	11	12	13	14	15	16	17
1. COVID-19 Health Anxiety	-																
2. COVID-19 Change in Negative Affect	−0.019	-															
3. Maladaptive Coping	−0.092	0.197	-														
4. Prosocial Coping	0.259	−0.090	0.084	-													
5. Distracted Coping	0.197	0.055	0.027	0.411	-												
6. School Engagement	0.213	−0.155	0.052	0.230	0.169	-											
7. Ethnic Identity Connection	0.244	−0.025	−0.004	0.265	0.192	0.142	-										
8. Ethnic Identity Achievement	0.265	−0.031	−0.002	0.285	0.177	0.151	0.775	-									
9. Awareness of Racism	0.195	0.064	0.042	0.187	0.117	0.120	0.611	0.677	-								
10. Trait Resilience	0.073	−0.128	-0.018	0.348	0.189	0.142	0.287	0.287	0.206	-							
11. Stressful Life Events	0.033	0.258	0.283	0.094	0.118	0.045	0.071	0.088	0.164	0.005	-						
12. School Difficulty	0.216	0.223	0.105	0.065	0.189	0.032	0.142	0.150	0.166	0.007	0.146	-					
13. Family Closeness	0.303	−0.087	−0.042	0.344	0.225	0.306	0.201	0.212	0.141	0.186	−0.013	0.142	-				
14. Peer Closeness	−0.052	−0.228	0.081	0.068	0.020	0.146	0.065	0.064	0.016	0.059	0.024	−0.132	0.007	-			
15. Change in Alcohol Use	−0.027	0.031	0.153	0.002	0.011	−0.032	0.014	0.020	0.005	0.000	0.067	0.018	0.006	0.036	-		
16. Change in Cannabis Use (Smoking)	−0.053	0.043	0.251	−0.015	0.005	0.003	−0.013	−0.007	−0.028	0.021	0.053	0.010	−0.045	0.017	0.247	-	
17. Change in Cannabis Use (Edibles)	−0.031	0.002	0.056	−0.004	0.019	−0.004	−0.010	−0.013	−0.024	0.048	−0.013	0.020	0.017	0.030	0.247	0.593	-

**Table 2:** Fit and Classification Criteria for One through Five Latent Profile Solutions.

Grade Group	7–9th						
# of Profiles	Converge Without Errors	-LL	CF	SABIC	Entropy	Smallest Profile %	Class Assignment Probability
1	Yes	−20,097.845	2.019	40,303.889	-	-	-
2	Yes	−19,560.895	2.04	39,287.852	0.686	35%	0.873/0.921
3	Yes	−19,138.897	1.81	38,501.919	0.801	7%	0.854/0.987/0.920
4	Yes	−18,861.622	1.65	38,005.334	0.796	7%	0.911/0.869/0.873/0.965
5	Yes	−18,757.901	1.83	37,855.855	0.766	7%	0.928/0.814/0.798/0.862/0.960
**Grade Group**	**10–12th**						
**# of Profiles**	**Converge** **Without Errors**	**-LL**	**CF**	**SABIC**	**Entropy**	**Smallest** **Profile %**	
1	Yes	−15,582.783	2.77	31,265.840	-	-	-
2	Yes	−15,082.770	2.18	30,319.532	0.751	34%	0.900/0.937
3	Yes	−14,753.500	1.84	29,714.718	0.816	6%	0.938/0.905/0.911
4	Yes	−14,628.352	1.74	29,518.131	0.823	6%	0.934/0.901/0.894/0.889
5	Yes	−14,528.806	1.59	29,372.758	0.847	2%	0.886/0.923/0.909/0.903/0.910

Note: Retained model highlighted in grey. -LL = negative loglikelihood; CF = Correction Factor; SABIC = Sample Size Adjusted Bayesian Information Criterion.

**Table 3:** Total and Profile-specific Sample Descriptives, Indicator Means, and Distal Means for Each Grade Group.

		7–9th *n* = 1142					10–12th, *n* = 860				
Ecosystemic Profiles		Average Risk and Resilience *n* = 588 (a)	High Resilience *n* = 394 (b)	Low Resilience *n* = 81 (c)	High Risk *n* = 77 (d)	Total	Average Risk and Resilience *n* = 379 (a)	High Resilience *n* = 330 (b)	Low Resilience *n* = 52 (c)	High Risk *n* = 99 (d)	Total
Categorical Descriptives *^i^*		*f (%)*	*f (%)*	*f (%)*	*f (%)*	*f (%)*	*f (%)*	*f (%)*	*f (%)*	*f (%)*	*f (%)*
Male		281 (47.79)	188 (47.72)	58 (71.60)	29 (37.66)	*556 (48.69)*	195 (51.45)	121 (36.67)	37 (71.15)	43 (43.43)	*396 (46.05)*
Female		307 (52.21)	206 (52.28)	23 (28.40)	48 (62.34)	*584 (51.23)*	184 (48.55)	209 (63.33)	15 (28.85)	56 (56.57)	*464 (53.95)*
Continuous Descriptives		M (SD)	M (SD)	M *(SD)*	M *(SD)*	M (SD)	M (SD)	M (SD)	M (SD)	M (SD)	M (SD)
Age		13.78 (1.14)	13.88 (0.97)	13.98 (1.16)	13.78 (1.02)	13.83 (1.08)	16.61 (0.96)	16.65 (0.99)	16.54 (1.39)	16.74 (0.90)	16.63 (1.0)
Grade		8.06 (0.85)	8.09 (0.82)	8.11 (0.86)	8.05 (0.78)	8.08 (0.83)	10.87 (0.82)	10.98 (0.82)	10.77 (0.78)	10.97 (0.76)	10.91 (0.82)
*Internal Vulnerability*											
COVID-19 Health Anxiety		2.55 (0.94) ^b,c^	3.26 (0.83) ^a,c,d^	2.18 (1.15) ^a,b,d^	2.56 (0.98) ^b,c^	2.78 (1.0)	2.35 (0.95) ^b,c^	3.11 (0.86) ^a,c,d^	2.0 (1.10) ^a,b,d^	2.35 (0.98) ^b,c^	2.61 (1.01)
Change in Negative Affect		3.17 (0.89) ^d^	3.02 (0.93) ^d^	3.06 (1.19) ^d^	3.66 (0.98) ^a,b,c^	3.15 (0.95)	3.04 (0.85) ^d^	3.25 (0.95)	3.39 (1.23)	3.42 (0.90) ^a^	3.19 (0.93)
COVID-19-SpecificMaladaptive Coping		1.11 (0.27) ^d^	1.07 (0.21) ^d^	1.17 (0.39) ^d^	3.08 (0.70) ^a,b,c^	1.24 (0.60)	1.27 (0.47) ^c,d^	1.22 (0.39) ^c,d^	1.62 (1.01) ^a,b,d^	3.18 (0.76) ^a,b,c^	1.51 (0.83)
*Internal Assets*											
COVID-19-Specific Prosocial Coping		2.11 (0.83) ^b,d^	2.79 (0.77) ^a,c^	1.92 (0.86) ^b,d^	2.56 (1.01) ^a,c^	2.36 (0.82)	2.30 (0.71) ^b,c,d^	2.78 (0.82) ^a,c^	1.74 (0.90) ^a,b,d^	2.61 (0.85) ^a,c^	2.48 (0.83)
COVID-19-Specific Distracted Coping		3.29 (1.03) ^b,c^	3.78 (0.93) ^a,c^	2.67 (1.27) ^a,b,d^	3.50 (0.98) ^c^	3.43 (1.06)	3.21 (1.01) ^b,c,d^	3.57 (0.96) ^a,c^	2.22 (1.16) ^a,b,d^	3.52 (0.94) ^a,c^	3.33 (1.04)
COVID-19-Specific] School Engagement		1.88 (0.82) ^b,c^	2.29 (0.94) ^a,c^	1.39 (0.61) ^a,b,d^	2.14 (0.93) ^c^	2.00 (0.89)	1.80 (0.85) ^c^	1.98 (0.89) ^c^	1.49 (0.82) ^a,b,d^	2.14 (0.97) ^c^	1.89 (0.89)
Ethnic Identity Connection		3.43 (0.63) ^b,c,d^	4.49 (0.51) ^a,c,d^	1.60 (0.68) ^a,b,d^	3.85 (0.93) ^a,b,c^	3.70 (0.98)	3.40 (0.61) ^b,c,d^	4.54 (0.49) ^a,c,d^	1.41 (0.51) ^a,b,d^	3.98 (0.75) ^a,b,c^	3.79 (0.97)
Ethnic Identity Achievement		3.25 (0.59) ^b,c,d^	4.38 (0.50) ^a,c,d^	1.47 (0.66) ^a,b,d^	3.57 (0.91) ^a,b,c^	3.55 (1.00)	3.23 (0.56) ^b,c,d^	4.51 (0.49) ^a,c,d^	1.40 (0.47) ^a,b,d^	3.99 (0.68) ^a,b,c^	3.71 (1.0)
Awareness of Racism		3.05 (0.72) ^b,c,d^	3.98 (0.79) ^a,c^	1.46 (0.66) ^a,b,d^	3.69 (0.89) ^a,c^	3.32 (1.01)	3.05 (0.70) ^b,c,d^	4.19 (0.73) ^a,c,d^	1.58 (0.72) ^a,b,d^	3.63 (0.81) ^a,b,c^	3.47 (1.01)
Trait Resilience		1.93 (0.86) ^b,c^	2.53 (0.84) ^a,c,d^	1.18 (1.19) ^a,b,d^	2.02 (1.12) ^b,c^	2.10 (0.97)	2.41 (0.80) ^b,c^	2.62 (0.85) ^a,c^	1.34 (1.13) ^a,b,d^	2.35 (0.90) ^c^	2.43 (0.89)
*External Adversity*											
Stressful Life Events		1.56 (1.50) ^d^	1.77 (1.69) ^d^	1.16 (1.69) ^d^	2.93 (1.92) ^a,b,c^	*1.70 (1.65)*	1.22 (1.36) ^d^	1.66 (1.46) ^d^	1.42 (1.46) ^d^	3.11 (1.85) ^a,b,c^	*1.62 (1.58)*
COVID-19-Specific School Challenges		2.50 (1.04) ^c,d^	2.53 (1.03) ^c,d^	1.85 (0.98) ^a,b,d^	2.92 (1.01) ^a,b,c^	*2.49 (1.05)*	2.26 (1.07) ^b,c,d^	2.77 (1.03) ^a,c^	1.95 (1.12) ^a,b,d^	2.85 (0.96) ^a,c^	*2.50 (1.08)*
*External Resources*											
COVID-19-Specific Family Closeness		2.11 (0.83) ^b,c^	2.75 (0.89) ^a,c,d^	1.79 (0.92) ^a,b,d^	2.23 (0.89) ^b,c^	*2.31 (0.92)*	1.94 (0.82) ^b,c^	2.43 (0.88) ^a,c,d^	1.52 (0.80) ^a,b,d^	2.18 (0.90) ^b,c^	*2.13 (0.89)*
COVID-19-Specific Peer Closeness		2.17 (0.82) ^b^	2.34 (0.87) ^a^	2.19 (0.76)	2.29 (0.89)	*2.24 (0.92)*	2.32 (0.81) ^d^	2.30 (0.84) ^d^	2.26 (0.88) ^d^	2.68 (0.85) ^a,b,c^	*2.35 (0.84)*
*Distal Outcomes*											
Change in Alcohol Use		−0.09 (0.54)	−0.13 (0.53)	−0.09 (0.64)	0.20 (1.17)	−*0.08 (0.61)*	−0.11 (0.65)	−0.08 (0.56)	−0.08 (0.76)	0.37 (1.08)	−*0.04 (0.70)*
Change in Cannabis Use: Smoking		−0.03 (0.49)	−0.12 (0.53)	0.07 (0.56)	0.41 (1.11)	−*0.02 (0.58)*	−0.37 (0.67)	−0.04 (0.71)	−0.06 (0.98)	0.46 (1.23)	*0.02 (0.81)*
Change in Cannabis Use: Edibles		−0.07 (0.47)	−0.10 (0.46)	0.01 (0.32)	0.22 (0.96)	−*0.06 (0.52)*	−0.07 (0.55)	−0.12 (0.56)	−0.25 (0.83)	−0.06 (0.78)	−*0.10 (0.61)*

Note: *^i^* Totals exclude missing data on descriptive questions. Superscript letters on indicator means indicate significant differences in the standardized means between referent profile and another profile at *p* < 0.05: a = Average Risk and Resilience; b = High Resilience; c = Low Resilience; d = High Risk.

**Table 4:** Mean Comparisons Between Ecosystemic Profiles of Substance use Changes During the COVID-19 Pandemic.

7–9th Grade			Average Risk and Resilience (a)		High Resilience (b)		Low Resilience (c)		High Risk (d)	
Distal Outcome	*χ* ^2^	*p*	*M*	*S.E.*	*M*	*S.E.*	*M*	*S.E.*	*M*	*S.E.*
Change in Alcohol Use	4.34	0.228	−0.089	0.027	−0.129	0.036	−0.087	0.066	0.197	0.137
Change in Cannabis Use (Smoking)	20.21	<0.001	−0.031 ^d^	0.029	−0.118 ^c,d^	0.031	0.070 ^b,d^	0.048	0.405 ^a,b,c^	0.150
Change in Cannabis Use (Edibles)	16.84	0.001	−0.074 ^d^	0.027	−0.103 ^d^	0.029	0.006	0.080	0.217 ^a,b^	0.097
**10–12th Grade**			**Average Risk** **and Resilience** **(a)**		**High Resilience** **(b)**		**Low Resilience** **(c)**		**High Risk** **(d)**	
Distal Outcome	*χ* ^2^	*p*	*M*	*S.E.*	*M*	*S.E.*	*M*	*S.E.*	*M*	*S.E.*
Change in Alcohol Use	34.45	<0.001	−0.112 ^d^	0.017	−0.078 ^d^	0.019	−0.076 ^d^	0.074	0.374 ^a,b,c^	0.101
Change in Cannabis Use (Smoking)	125.33	<0.001	−0.050 ^d^	0.036	−0.049 ^d^	0.071	−0.060 ^d^	0.147	0.528 ^a,b,c^	0.066
Change in Cannabis Use (Edibles)	3.63	0.304	−0.066	0.044	−0.124	0.044	−0.066	0.044	−0.055	0.078

Note. Letter superscripts indicate profile is significantly different (*p* < 0.05) than a different profile labeled according to the specified superscript: a = Average Risk and Resilience; b = High Resilience; c = Low Resilience; d = High Risk.

## Data Availability

The data presented in this study are available on request from the corresponding author. These data are not yet available within a public repository because data collection for the larger OYOF survey is currently in progress.

## Appendix A

**Table A1:** Indicators of Ecosystemic Risk and Resilience.

Internal Vulnerabilities	Cronbach’s Alpha	Scale
*COVID-19 Health Anxiety: How worried are you about…*	0.915	1 = Not worried 2 = A little worried 3 = Worried 4 = Very worried
…getting COVID-19?		
…long-term health problems if you get COVID-19?		
…a family member getting COVID-19?		
…dying from COVID-19?		
…a family member dying from COVID-19?		
…giving someone else COVID-19?		
*Change in Negative Affect: Compared to before COVID-19, are you…*	0.923	1 = Much less 2 = Less 3 = About the same 4 = More 5 = Much more
...more or less sad now?		
...more or less lonely now?		
...more or less bored now?		
...more or less depressed now?		
...more or less angry now?		
...more or less worried now?		
...more or less anxious now?		
...having more or less trouble sleeping now?		
...more or less interested in normal activities?		
...having more or less trouble concentrating?		
*COVID-19-Specific Maladaptive Coping: How often have you done each of the following to deal with your stress related to COVID-19?*	0.811	1 = Never 2 = Not very often 3 = Sometimes 4 = Often 5 = Very often
Drinking alcohol		
Using tobacco (smoking, chewing or vaping)		
Using marijuana (smoking, edibles, vaping)		
Using other illegal drugs		
**Internal Assets**		
*COVID-19-Specific Prosocial Coping:* *How often have you done each of the following to deal with your stress related to COVID-19?*	0.623	1 = Never 2 = Not very often 3 = Sometimes 4 = Often 5 = Very often
Meditation/Mindfulness practices/Prayer		
Exercising		
Joining in family activities like games or sport		
Talking to someone about my stress (parents, school counselors, or doctors)		
Volunteer work in my community		
COVID-19-Specific Distracted Coping: How often have you done each of the following to deal with your stress related to COVID-19?	0.627	1 = Never 2 = Not very often 3 = Sometimes 4 = Often 5 = Very often
Talking with friends virtually		
Using social media		
Watching TV/Playing video games		
*COVID-19-Specific School Engagement: Please rate how true each statement below is for you:*	0.761	1 = Not at all true 2 = A little true 3 = True 4 = Very true
I attend school more (either remotely or in person) than before COVID-19.		
I am getting better grades now than before COVID-19.		
I am enjoying school more now than before COVID-19.		
*Ethnic Identity Connection: For each statement below, say how close it is to your opinion using the following scale:*	0.776	1 = Strongly disagree 2 = Disagree 3 = Neither agree nor disagree 4 = Agree 5 = Strongly agree
It is important to me to think of myself as American Indian		
I feel that I am part of the American Indian community.		
I feel close to others in the American Indian community.		
I have a lot of pride in what members of the American Indian community have achieved.		
*Ethnic Identity Achievement: For each statement below, say how close it is to your opinion using the following scale:*	0.798	1 = Strongly disagree 2 = Disagree 3 = Neither agree nor disagree 4 = Agree 5 = Strongly agree
It helps me when others in the American Indian community are successful.		
It is important for my family and the American Indian community that I succeed in school.		
If I work hard and get good grades, other American Indian people will respect me.		
If I am successful, it will help the American Indian community.		
*Awareness of Racism: For each statement below, say how close it is to your opinion using the following scale:*	0.713	1 = Strongly disagree 2 = Disagree 3 = Neither agree nor disagree 4 = Agree 5 = Strongly agree
As an American Indian, the way I look and speak influences what others expect of me.		
Some people will treat me differently because I am American Indian.		
People might have negative ideas about my abilities because I am American Indian.		
Things in the American Indian community are not as good as they could be because of lack of opportunity.		
*Trait Resilience: Please rate how much you agree with the following statements as they apply to you over the past month.*	0.887	0 = Not at all true 1 = Rarely true 2 = Sometimes true 3 = Often true 4 = True nearly all of the time
I am able to adapt when changes occur.		
Under pressure, I stay focused and think clearly.		
I am not easily discouraged by failure.		
I try to see the humorous side of things when I am faced with problems.		
I tend to bounce back after illness, injury or other hardships.		
I think of myself as a strong person when dealing with life’s challenges and difficulties.		
I can deal with whatever comes my way.		
Having to cope with stress can make me stronger.		
I believe I can achieve my goals, even if there are obstacles.		
I am able to handle unpleasant or painful feelings like sadness, fear, and anger.		
I am able to adapt when changes occur.		
Under pressure, I stay focused and think clearly.		
I am not easily discouraged by failure.		
**External Adversity**		
*Stressful Life Events: In the past 12 months, did any of the following events happen to you?*	NA	0 = No 1 = Yes
Entered school as a new or transfer student.		
Had a parent or guardian be unable to find employment.		
Found out people are gossiping about you.		
Had a friend attempt suicide.		
Had a serious argument with a friend.		
Broke up with a boyfriend/girlfriend or significant other.		
Was in a serious car wreck.		
Entered school as a new or transfer student.		
*COVID-19-Specific School Challenges: Please rate how true each statement below is for you:*	0.826	1 = Not at all true 2 = A little true 3 = True 4 = Very true
It is harder for me to focus on my schoolwork.		
I am falling behind in my schoolwork more.		
I am more worried about school.		
**External Resources**		
*COVID-19-Specific Family Closeness: Please rate how true each statement below is for you:*	0.706	1 = Not at all true 2 = A little true 3 = True 4 = Very true
I spend more time with my family.		
My family is closer.		
My parents/guardians supervise my activities more.		
*COVID-19-Specific Peer Closeness: Please rate how true each statement below is for you:*	0.573 ^a^	1 = Not at all true 2 = A little true 3 = True 4 = Very true
I see my friends more in person since COVID-19 began.		
I feel closer to my friends since COVID-19 began.		
**Distal Outcomes**		
After COVID-19 started, how much did your use of alcohol change?	NA	−2 = Decreased a lot −1 = Decreased a little 0 = No change 1 = Increased a little 2 = Increased a lot
After COVID-19 started, how much did your smoking of marijuana change?	NA	
After COVID-19 started, how much did your use of marijuana edibles change?	NA	

^a^: This is a bivariate correlation (r).

## References

[1] Jones E., Mitra A., Bhuiyan A. (2021). Impact of COVID-19 on Mental Health in Adolescents: A Systematic Review. Int. J. Environ. Res. Public Health.

[2] Richter L. (2020). The Effects of the COVID-19 Pandemic on the Risk of Youth Substance Use. J. Adolesc. Health.

[3] Lee J. (2020). Mental health effects of school closures during COVID-19. Lancet Child Adolesc. Health.

[4] Swaim R.C., Stanley L.R., Meich R.A., Patrick M.E., Crabtree M.A., Prince M.A. (2022). A Comparison of COVID-19 outcomes between reservation-based American Indian and U.S. national students. Am. J. Prev. Med. Focus.

[5] Bear C.R., Terrill W.P.A., Frates A., Peterson P., Ulrich J. (2021). Challenges for Rural Native American Students with Disabilities during COVID-19. Rural Speéc. Educ. Q..

[6] Swaim R.C., Stanley L.R. (2018). Substance Use Among American Indian Youths on Reservations Compared with a National Sample of US Adolescents. JAMA Netw. Open.

[7] Fergus S., Zimmerman M.A. (2005). Adolescent Resilience: A Framework for Understanding Healthy Development in the Face of Risk. Annu. Rev. Public Health.

[8] Waller M.A. (2001). Resilience in ecosystemic context: Evolution of the concept. Am. J. Orthopsychiatry.

[9] Raifman M.A., Raifman J.R. (2020). Disparities in the Population at Risk of Severe Illness From COVID-19 by Race/Ethnicity and Income. Am. J. Prev. Med..

[10] Gawthrop E. The Color of Coronavirus: COVID-19 Deaths by Race and Ethnicity in the U.S. https://www.ywboston.org/wp-content/uploads/2021/07/Color-of-Coronavirus_-COVID-19-deaths-a...-race-and-ethnicity-%E2%80%94-APM-Research-Lab.pdf.

[11] Burki T. (2021). COVID-19 among American Indians and Alaska Natives. Lancet Infect. Dis..

[12] Wang H. (2021). Why the Navajo Nation was hit so hard by coronavirus: Understanding the disproportionate impact of the COVID-19 pandemic. Appl. Geogr..

[13] Hathaway E.D. (2020). American Indian and Alaska Native People: Social Vulnerability and COVID-19. J. Rural Health.

[14] Household Experiences in America during the Delta Variant Outbreak by Race/Ethnicity 2021. https://www.rwjf.org/en/library/research/2021/10/household-experiences-in-america-during-the-delta-variant-outbreak.html.

[15] Maudrie T.L., Lessard K.H., Dickerson J., Aulandez K.M.W., Barlow A., O’Keefe V.M. (2021). Our Collective Needs and Strengths: Urban AI/ANs and the COVID-19 Pandemic. Front. Sociol..

[16] LaFromboise T.D., Hoyt D., Oliver L., Whitbeck L.B. (2006). Family, community, and school influences on resilience among American Indian adolescents in the upper midwest. J. Community Psychol..

[17] Doshi S., Jordan A., Kelly K., Solomon D. (2020). The COVID-19 Response in Indian Country: A Federal Failure. https://www.americanprogress.org/article/covid-19-response-indian-country/.

[18] Powder J. Keys to the Navajo Nation’s COVID-19 Vaccination Success. https://publichealth.jhu.edu/2021/keys-to-the-navajo-nations-covid-19-vaccination-success.

[19] Scott S.R., Rivera K.M., Rushing E., Manczak E.M., Rozek C.S., Doom J.R. (2020). “I Hate This”: A Qualitative Analysis of Adolescents’ Self-Reported Challenges During the COVID-19 Pandemic. J. Adolesc. Health.

[20] Hawke L.D., Barbic S.P., Voineskos A., Szatmari P., Cleverley K., Hayes E., Relihan J., Daley M., Courtney D., Cheung A. (2020). Impacts of COVID-19 on youth mental health, substance use, and well-being: A rapid survey of clinical and community samples. Can. J. Psychiatry.

[21] Magson N.R., Freeman J.Y.A., Rapee R.M., Richardson C.E., Oar E.L., Fardouly J. (2020). Risk and Protective Factors for Prospective Changes in Adolescent Mental Health during the COVID-19 Pandemic. J. Youth Adolesc..

[22] D’Amico E.J., Palimaru A.I., Dickerson D.L., Dong L., Brown R.A., Johnson C.L., Klein D.J., Troxel W.M. (2020). Risk and resilience factors in Urban American Indian and Alaska Native youth during the coronavirus pandemic. Am. Indian Cult. Res. J..

[23] Stanley L.R., Swaim R.C., Smith J.K., Conner B.T. (2020). Early onset of cannabis use and alcohol intoxication predicts prescription drug misuse in American Indian and non-American Indian adolescents living on or near reservations. Am. J. Drug Alcohol Abus..

[24] Stanley L.R., Harness S.D., Swaim R.C., Beauvais F. (2014). Rates of Substance Use of American Indian Students in 8th, 10th, and 12th Grades Living on or near Reservations: Update, 2009–2012. Public Health Rep..

[25] Ungar M., Theron L., Murphy K., Jefferies P. (2021). Researching Multisystemic Resilience: A Sample Methodology. Front. Psychol..

[26] Beebe L.A., Vesely S.K., Oman R.F., Tolma E., Aspy C.B., Rodine S. (2008). Protective Assets for Non-use of Alcohol, Tobacco and Other Drugs among Urban American Indian Youth in Oklahoma. Matern. Child Health J..

[27] Henson M., Sabo S., Trujillo A., Teufel-Shone N. (2016). Identifying Protective Factors to Promote Health in American Indian and Alaska Native Adolescents: A Literature Review. J. Prim. Prev..

[28] Ungar M., Jefferies P. (2021). Becoming More Rugged and Better Resourced: The R2 Resilience Program’s© Psychosocial Approach to Thriving. Front. Psychol..

[29] Ungar M. (2018). Systemic resilience: Principles and processes for a science of change in contexts of adversity. Ecol. Soc..

[30] Ungar M. (2006). Resilience across Cultures. Br. J. Soc. Work.

[31] Stiffman A.R., Brown E., Freedenthal S., House L., Ostmann E., Yu M.S. (2007). American Indian Youth: Personal, Familial, and Environmental Strengths. J. Child Fam. Stud..

[32] Braverman M.T. (2001). Applying resilience theory to the prevention of adolescent substance abuse Author. Cent. Youth Dev. FOCUS.

[33] Liang L., Ren H., Cao R., Hu Y., Qin Z., Li C., Mei S. (2020). The Effect of COVID-19 on Youth Mental Health. Psychiatr. Q..

[34] Dumas T.M., Ellis W., Litt D.M. (2020). What Does Adolescent Substance Use Look Like during the COVID-19 Pandemic? Examining Changes in Frequency, Social Contexts, and Pandemic-Related Predictors. J. Adolesc. Health.

[35] Acuff S.F., Tucker J.A., Murphy J.G. (2021). Behavioral economics of substance use: Understanding and reducing harmful use during the COVID-19 pandemic. Exp. Clin. Psychopharmacol..

[36] Ellis W.E., Dumas T.M., Forbes L.M. (2020). Physically isolated but socially connected: Psychological adjustment and stress among adolescents during the initial COVID-19 crisis. Can. J. Behav. Sci. Rev. Can. Sci. Comport..

[37] Howard M.C., Hoffman M.E. (2017). Variable-Centered, Person-Centered, and Person-Specific Approaches. Organ. Res. Methods.

[38] Neblett E.W., Sosoo E.E., Willis H.A., Bernard D.L., Bae J., Billingsley J.T. (2016). Racism, Racial Resilience, and African American Youth Development. Adv. Child Dev. Behav..

[39] Prince M.A., Fidler D.J. (2021). Analytic approaches to heterogeneity in neurogenetic syndrome research. Int. Rev. Res. Dev. Disabil..

[40] Ferguson S.L., Moore E.W.G., Hull D.M. (2019). Finding latent groups in observed data: A primer on latent profile analysis in Mplus for applied researchers. Int. J. Behav. Dev..

[41] Ladouceur C.D. COVID-19 Adolescent Symptom & Psychological Experience Questionnaire (CASPE) 2020. https://www.phenxtoolkit.org/toolkit_content/PDF/CASPE_Adolescent.pdf.

[42] The Coronavirus Health Impact Survey (CRISIS) V0.3, Youth Self-Report Baseline Form: Current Form 2020. https://www.phenxtoolkit.org/toolkit_content/PDF/CRISIS_Baseline_Youth.pdf.

[43] Campbell-Sills L., Stein M.B. (2007). Psychometric analysis and refinement of the connor–davidson resilience scale (CD-RISC): Validation of a 10-item measure of resilience. J. Trauma. Stress.

[44] Goins R.T., Gregg J.J., Fiske A. (2012). Psychometric Properties of the Connor-Davidson Resilience Scale with Older American Indians. Res. Aging.

[45] Jorgensen I.E., Seedat S. (2008). Factor structure of the Connor-Davidson Resilience Scale in South African adolescents. Int. J. Adolesc. Med. Health.

[46] Oyserman D., Brickman D., Rhodes M., Fuligni A. (2007). Racial-Ethnic Identity: Content and consequences for African American, Latino, and Latina youths. Contesting Stereotypes and Creating Identities: Social Categories, Social Identities, and Educational Participation.

[47] Baldwin J.A., Brown B.G., Wayment H., Nez R.A., Brelsford K. (2011). Culture and Context: Buffering the Relationship between Stressful Life Events and Risky Behaviors in American Indian Youth. Subst. Use Misuse.

[48] Graham J.W., Taylor B.J., Olchowski A.E., Cumsille P.E. (2006). Planned missing data designs in psychological research. Psychol. Methods.

[49] Enders C.K. (2022). Applied Missing Data Analysis.

[50] Muthén L.K., Muthen L.K., Muthen B.O. (2017). Mplus User’s Guide.

[51] Enders C.K., Bandalos D.L. (2001). The Relative Performance of Full Information Maximum Likelihood Estimation for Missing Data in Structural Equation Models. Struct. Equ. Model..

[52] Croux C., Dhaene G., Hoorelbeke D. (2004). Robust standard errors for robust estimators. CES-Discuss. Pap. Ser..

[53] Tein J.-Y., Coxe S., Cham H. (2013). Statistical Power to Detect the Correct Number of Classes in Latent Profile Analysis. Struct. Equ. Model. A Multidiscip. J..

[54] Eid M., Langeheine R., Diener E. (2003). Comparing Typological Structures Across Cultures By Multigroup Latent Class Analysis. J. Cross-Cultural Psychol..

[55] Asparouhov T., Muthén B. (2014). Auxiliary Variables in Mixture Modeling: Three-Step Approaches Using Mplus. Struct. Equ. Model. Multidiscip. J..

[56] Satorra A., Bentler P.M. (2010). Ensuring Positiveness of the Scaled Difference Chi-square Test Statistic. Psychometrika.

[57] Miller-Graff L.E. (2020). The Multidimensional Taxonomy of Individual Resilience. Trauma Violence Abus..

[58] Malhi G.S., Das P., Bell E., Mattingly G., Mannie Z. (2019). Modelling resilience in adolescence and adversity: A novel framework to inform research and practice. Transl. Psychiatry.

[59] Chen S., Bonanno G.A. (2020). Psychological adjustment during the global outbreak of COVID-19: A resilience perspective. Psychol. Trauma Theory Res. Pract. Policy.

[60] PeConga E.K., Gauthier G.M., Holloway A., Walker R.S.W., Rosencrans P.L., Zoellner L.A., Bedard-Gilligan M. (2020). Resilience is spreading: Mental health within the COVID-19 pandemic. Psychol. Trauma Theory Res. Pract. Policy.

[61] Bonanno G.A., Mancini A.D. (2012). Beyond resilience and PTSD: Mapping the heterogeneity of responses to potential trauma. Psychol. Trauma Theory Res. Pract. Policy.

[62] Galatzer-Levy I.R., Huang S.H., Bonanno G.A. (2018). Trajectories of resilience and dysfunction following potential trauma: A review and statistical evaluation. Clin. Psychol. Rev..

[63] Tingey L., Cwik M.F., Rosenstock S., Goklish N., Larzelere-Hinton F., Lee A., Suttle R., Alchesay M., Massey K., Barlow A. (2016). Risk and protective factors for heavy binge alcohol use among American Indian adolescents utilizing emergency health services. Am. J. Drug Alcohol Abus..

[64] McKinley C.E., Lilly J. (2021). “It’s in the family circle”: Communication promoting Indigenous family resilience. Fam. Relat..

[65] Martin D., Yurkovich E. (2013). “Close-Knit” Defines a Healthy Native American Indian Family. J. Fam. Nurs..

[66] Tajfel H., Turner J.C., Austin W.G., Worchel S. (1979). An Integrative Theory of Intergroup Conflict. The Social Psychology of Intergroup Relations.

[67] Brewer M.B., Gardner W. (1996). Who is this "We"? Levels of collective identity and self representations. J. Pers. Soc. Psychol..

[68] Hogg M.A. (2000). Social Identity and Social Comparison. Handbook of Social Comparison.

[69] Altschul I., Oyserman D., Bybee D. (2006). Racial-Ethnic Identity in Mid-Adolescence: Content and Change as Predictors of Academic Achievement. Child Dev..

[70] Crocker J., Major B. (1989). Social stigma and self-esteem: The self-protective properties of stigma. Psychol. Rev..

[71] Smokowski P.R., Evans C.B.R., Cotter K.L., Webber K.C. (2013). Ethnic Identity and Mental Health in American Indian Youth: Examining Mediation Pathways Through Self-esteem, and Future Optimism. J. Youth Adolesc..

[72] Tyser J., Scott W.D., Readdy T., McCrea S.M. (2013). The Role of Goal Representations, Cultural Identity, and Dispositional Optimism in the Depressive Experiences of American Indian Youth from a Northern Plains Tribe. J. Youth Adolesc..

[73] Brown R.A., Dickerson D.L., D’Amico E.J. (2016). Cultural Identity Among Urban American Indian/Alaska Native Youth: Implications for Alcohol and Drug Use. Prev. Sci..

[74] Kulis S., Napoli M., Marsiglia F.F. (2002). Ethnic pride, biculturalism, and drug use norms of urban American Indian adolescents. Soc. Work Res..

[75] Dryhurst S., Schneider C.R., Kerr J., Freeman A.L.J., Recchia G., van der Bles A.M., Spiegelhalter D., van der Linden S. (2020). Risk perceptions of COVID-19 around the world. J. Risk Res..

[76] Vinayak S., Judge J. (2018). Resilience and empathy as predictors of psychological wellbeing among adolescents. Int. J. Health Sci. Res..

[77] Whitesell N.R., Mitchell C.M., Kaufman C.E., Spicer P., The Voices of Indian Teens Project Team (2006). Developmental Trajectories of Personal and Collective Self-Concept among American Indian Adolescents. Child Dev..

[78] Owen M.J., Sundberg M.A., Dionne J., Kosobuski A.W. (2021). The Impact of COVID-19 on American Indian and Alaska Native Communities: A Call for Better Relational Models. Am. J. Public Health.

[79] Heart M.Y.H.B., Debruyn L.M. (1998). The American Indian Holocaust: Healing historical unresolved grief. Am. Indian Alsk. Nativ. Ment. Health Res..

[80] Evans W., Davis B. (2018). Exploring the Relationship between Sense of Coherence and Historical Trauma among American Indian Youth. Am. Indian Alsk. Nativ. Ment. Health Res..

[81] Whitesell N.R., Mitchell C.M., Spicer P., Voices of Indian Teens Project Team (2009). A longitudinal study of self-esteem, cultural identity, and academic success among American Indian adolescents. Cult. Divers. Ethn. Minor. Psychol..

[82] Galliher R.V., Jones M.D., Dahl A. (2011). Concurrent and longitudinal effects of ethnic identity and experiences of discrimination on psychosocial adjustment of Navajo adolescents. Dev. Psychol..

[83] Graham B.L. (2001). Resilience among American Indian Youth: First Nation’s Youth Resilience Study.

[84] Sayers J.F., MacDonald K.A., Fiske J.-A., Newell M., George E., Cornet W. (2001). First Nations Women, Governance and the Indian Act: A Collection of Policy Research Reports.

[85] Heart M.Y.H.B., Elkins J., Tafoya G., Bird D., Salvador M. (2012). Wicasa Was’aka: Restoring the Traditional Strength of American Indian Boys and Men. Am. J. Public Health.

[86] White J., Godfrey J., Moccasin B. (2006). American Indian Fathering in the Dakota Nation: Use of Akicita as a Fatherhood Standard. Father. J. Theory Res. Pract. Men Father..

[87] Ostaszewski K., Zimmerman M.A. (2006). The Effects of Cumulative Risks and Promotive Factors on Urban Adolescent Alcohol and Other Drug Use: A Longitudinal Study of Resiliency. Am. J. Community Psychol..

[88] Ungar M. (2004). A Constructionst Discourse on resilience: Multiple contexts, multiple realities among at-risk children and youth. Youth Soc..

[89] Miech R., Patrick M.E., Keyes K., O’Malley P.M., Johnston L. (2021). Adolescent drug use before and during U.S. national COVID-19 social distancing policies. Drug Alcohol Depend..

